# Consumption of a diet rich in *Brassica* vegetables is associated with a reduced abundance of sulphate‐reducing bacteria: A randomised crossover study

**DOI:** 10.1002/mnfr.201600992

**Published:** 2017-04-12

**Authors:** Lee Kellingray, Henri S. Tapp, Shikha Saha, Joanne F. Doleman, Arjan Narbad, Richard F. Mithen

**Affiliations:** ^1^ Food and Health Programme Institute of Food Research Norwich Research Park Norwich UK; ^2^ Analytical Sciences Unit Institute of Food Research Norwich Research Park Norwich UK; ^3^ Gut Health and Food Safety Programme Institute of Food Research Norwich Research Park Norwich UK

**Keywords:** *Brassica*, Gastrointestinal health, Gut microbiota, Randomised crossover study, Sulphate‐reducing bacteria

## Abstract

**Scope:**

We examined whether a *Brassica*‐rich diet was associated with an increase in the relative abundance of intestinal lactobacilli and sulphate‐reducing bacteria (SRB), or alteration to the composition of the gut microbiota, in healthy adults.

**Methods and results:**

A randomised crossover study was performed with ten healthy adults who were fed a high‐ and a low‐*Brassica* diet for 2‐wk periods, with a 2‐wk washout phase separating the diets. The high‐*Brassica* diet consisted of six 84 g portions of broccoli, six 84 g portions of cauliflower and six 300 g portions of a broccoli and sweet potato soup. The low‐*Brassica* diet consisted of one 84 g portion of broccoli and one 84 g portion of cauliflower. Faecal microbiota composition was measured in samples collected following 2‐wk *Brassica*‐free periods (consumption of all *Brassica* prohibited), and after each diet, whereby the only *Brassica* consumed was that supplied by the study team. No significant changes to the relative abundance of lactobacilli were observed (*p* = 0.8019). The increased consumption of *Brassica* was associated with a reduction in the relative abundance of SRB (*p* = 0.0215), and members of the *Rikenellaceae*, *Ruminococcaceae*, *Mogibacteriaceae*, *Clostridium* and unclassified *Clostridiales* (*p* < 0.01).

**Conclusion:**

The increased consumption of *Brassica* vegetables was linked to a reduced relative abundance of SRB, and therefore may be potentially beneficial to gastrointestinal health.

**Abbreviations**
ANOVA: analysis of variance
GI: gastrointestinal
H_2_S: hydrogen sulphide
QIIME: Quantitative Insights into Microbial Ecology
rRNA: ribosomal RNA
SRB: sulphate‐reducing bacteria

## 1 Introduction

There is a growing body of largely epidemiological evidence that suggests that diets rich in *Brassica* vegetables may be associated with multiple health benefits, including a reduction in both the incidence and progression of cancers 1, 2. While many potential mechanisms have been proposed that may underpin the health‐promoting properties of *Brassica* vegetables 3, there have been relatively few studies on how these sulphur‐rich vegetables may modulate the gut microbiota 4, 5. Certain dietary interventions have been shown to increase the frequency of *Lactobacillus* 6, 7, a change that would be considered to be largely beneficial to health. Fermented *Brassica* food products, such as sauerkraut and kimchi, are particularly rich in *Lactobacillus*, and a diet rich in *Brassica* may promote *Lactobacillus* growth in the colon. In contrast, sulphate‐rich vegetables such as *Brassica* may promote the growth of sulphate‐reducing bacteria (SRB) that may have potential negative effects on gastrointestinal (GI) health. SRB are members of the human gut microbiota that are able to perform dissimilatory sulphate reduction; the reduction of sulphate coupled with the oxidation of dihydrogen, leading to the production of hydrogen sulphide (H_2_S) 8. Increases in sulphide and the relative abundance of SRB have been reported in the faeces of sufferers of ulcerative colitis and irritable bowel syndrome 9, 10, 11, and increased levels of H_2_S have been linked to multiple GI disorders 12, 13, 14. However, at low levels, the importance of H_2_S as a gasotransmitter required for the maintenance of cell and tissue health, along with nitric oxide and carbon monoxide, is being increasingly realised 15, 16. Thus, understanding the genetic and environmental factors that determine the extent of SRB in the gut microbiota may be of importance in both healthy people and sufferers of ulcerative colitis and irritable bowel syndrome.

*Brassica* vegetables are a significant source of inorganic sulphate in the diet 17, and it is conceivable that diets rich in these vegetables may encourage the growth of SRB, with potentially negative health consequences due to the pro‐inflammatory activity of H_2_S. Thus, it is important to investigate whether any reduction in cancer‐related risk may be accompanied with an additional prebiotic effect on the numbers of lactobacilli, or offset by potentially negative effects on GI health due to an enhancement of SRB.

The primary aim of this study is to investigate whether a high‐*Brassica* diet is associated with an increased relative abundance of members of the genus *Lactobacillus* in healthy adults. The secondary aims are to identify whether the consumption of *Brassica* vegetables is associated with changes to the relative abundance of SRB, or an alteration to the community structure of the microbiota in the human GI tract.

## 2 Materials and methods

### 2.1 Study participants

Ten men and women aged between 18 and 50 years old, with a BMI between 19.5 and 30 kg/m^2^, were recruited from Norwich, UK, into the *Brassica* dietary study, which was run at the Institute of Food Research, Norwich. The primary outcome of the study was the difference in abundance of members of *Lactobacillus* in faecal samples collected following the low‐*Brassica* and high‐*Brassica* interventions within a randomised two‐way crossover study. Based on data presented in Table 1 of Tannock et al. 18, for a log‐transformed response, assuming a standardised mean difference of 1.0, then to reach a 5% significance level with 80% power would require ten subjects to complete the study. The exclusion criteria included a medical history of GI disorders/surgery, long‐term medical conditions (e.g., diabetes) or those taking medication, such as laxatives, that would have affected the study outcome, recent (within past 3 months) or long‐term antibiotic use, intermittent prebiotic and/or probiotic usage, regular use of over‐the‐counter medications for GI‐associated conditions, those on a diet plan that may have affected the study outcome, suffered gastric symptoms during or following overseas travel, had experienced blood in stools that was undiagnosed by a medical practitioner or typically produced stools characterised as types 1, 2 or 7 by the Bristol Stool Chart. Demographic information was collected and health questionnaires were completed during the eligibility screenings. The study was approved by the Institute of Food Research Human Research Governance committee (IFR04/2014) and Norfolk Research Ethics Committee (14/EE/1078). The informed consent of all participating subjects was obtained, and the trial is registered at http://www.clinicaltrials.gov (NCT02291328).

**Table 1:** Age, gender, body weight, BMI and smoking status of the study participants

Participant code	Age (years)	Gender	Body weight (kg)	BMI (kg/m^2^)	Smoker
1	35	Male	84.9	28.5	N
2	25	Female	68.1	23.5	N
3	25	Female	72.3	24.8	N
4	32	Female	67.1	21.1	N
5	39	Female	51.1	20.3	N
6	40	Male	81.7	24.4	N
7	36	Male	98.1	26.0	N
8	41	Female	71.5	25.3	Y
9	28	Male	81.4	27.5	N
10	34	Female	57.9	23.8	N

### 2.2 Dietary intervention

The study was a randomised, two‐phase crossover intervention study, with one phase representing a low‐*Brassica* diet, and the other phase a high‐*Brassica* diet, separated by a minimum 2‐wk washout period. Following an initial 2‐wk period in which the participants avoided the consumption of *Brassica* and other foods that contain glucosinolates, the participants were allocated to either low–high or high–low *Brassica* diets. The low‐*Brassica* diet consisted of one portion of 84 g frozen broccoli and one portion of 84 g frozen cauliflower, purchased from a Sainsbury's supermarket (J Sainsbury PLC, UK), and a single portion of the participant's choice was to be consumed each week for 2 wk. The high‐*Brassica* diet consisted of six 84 g portions of frozen broccoli, six 84 g portions of frozen cauliflower and six 300 g portions of a broccoli and sweet potato soup containing 84 g broccoli (Bakkavor Group Ltd, UK). Three portions of frozen broccoli, three portions of frozen cauliflower and three broccoli and sweet potato soups were consumed each week, for a period of 2 wk. The diets were supplied in insulated cool bags, and arrangements were made in such a way that there was no risk of thawing before they could be stored in the participants’ freezer. The participants were provided with clear written instructions on how the vegetables and soups were to be stored and prepared. Vegetable portions were steamed for 5 min on the top tier of a pre‐heated steamer that had been supplied to the participants, and the soups were cooked from chilled on an oven hob at medium heat for 4 min, or in a microwave (750 W) for 5 min. During the study period, which included a 2‐wk period prior to each intervention phase, participants were required to not consume *Brassica* and other foods that contain glucosinolates, with the exception of the diets provided for the intervention phases. Participants were asked to complete stool charts for consecutive 7‐day periods during each intervention phase, and in the 2‐wk period following completion of the second intervention phase, and these indicated no adverse effects on gut function of any participants during the study period.

### 2.3 Diet analysis

Representative portions of the cauliflower and broccoli were steamed from frozen for 5 min, and the broccoli and sweet potato soups were microwaved (750 W) from frozen for 8 min and 30 s, prior to being lyophilised and ground to a fine powder, in preparation for the following analyses.

#### 2.3.1 Total sulphur measurements

An aliquot (∼1.5 g) of each of the powdered foods were sent to Eurofins Food Testing UK Ltd to measure the amount of total sulphur. Inductively Coupled Plasma Spectrophotometry Optical Emission Spectrometry (Agilent 700 series) was performed following an acid digestion step using nitric acid and hydrochloric acid.

#### 2.3.2 Sulphate anion analysis

Aliquots (25 mg) of washed and milled polyvinylpolypirrolidone were mixed with 1 mL of ultrapure water and stored overnight at 4°C. A known mass (20–30 mg) of each of the frozen powdered foods were added to a tube containing the soaked polyvinylpolypirrolidone, and shaken for 1 h at 4°C. The samples were maintained at 95°C for 15 min, centrifuged at 4000 × *g* for 15 min at 4°C and the supernatant was filtered through a PVDF 0.45 μm syringe filter into autosampler vials for LC‐MS. The sulphate anion was analysed using an Acclaimۛ Trinityۛ P1 3 μm (3 × 50 mm) column connected to an 1100 series Single Quad LC‐MS system (Agilent Technologies) composed of a 1200 series degasser, binary pump, cooled autosampler, column oven, DAD and mass spectrometer (G1956B, Agilent Technologies). The samples were eluted at 0.5 mL/min with a gradient of increasing ammonium acetate: solvent A (200 mM ammonium acetate adjusted to pH 4 with 0.1 M acetic acid), solvent B (60% ACN/40% ultrapure water). The gradient started at 5% solvent A increasing over 15 min to 90% solvent A and finally re‐equilibrated to 5% solvent A for 5 min. The LC eluent flow was sprayed into the mass spectrometer interface without splitting. Sulphate was monitored using MS in selective ion monitor mode (*m/z* = 97) in negative polarity with ESI. The quantification was performed using matrix match calibration curve. Identification was achieved on the basis of retention time, and quantification was performed through the use of calibration standards.

#### 2.3.3 Glucosinolate analysis

Glucosinolates were converted to the equivalent desulphoglucosinolates prior to being measured using HPLC as described by Magrath et al. 19.

#### 2.3.4 *S*‐methylcysteine sulphoxide analysis

The extraction of *S*‐methylcysteine sulphoxide was performed as described by Bernaert et al. 20, with the supernatants diluted 1:10 using HPLC grade water containing 0.1% formic acid. Analysis was performed by LC‐MS/MS using an Agilent SB‐AQ 1.8 μm (100 × 2.1 mm) C18 column, with an Agilent Zorbax guard column, connected to a model 1290 infinity 6490 Triple Quad LC‐MS/MS system (Agilent Technologies) with a 6490 mass spectrometer.

### 2.4 Faecal sample collection

Each of the ten participants produced faecal samples at the following stages: the day before commencing the diet restriction, immediately prior to the two intervention phases, the day after each completed intervention phase and 2 wk after the diet restriction ceased. Participants were provided with faecal collection kits, which included a stool collection bag and an ice pack. They were asked to defecate directly into the bag, which was secured and placed with the ice pack into an insulated container, and delivered to the study scientist. The samples were then homogenised by physical manipulation before aliquots were taken and stored at −80°C.

### 2.5 Faecal bacterial DNA extraction

Faecal bacterial genomic DNA was extracted from 200 mg of faecal samples using a FastDNA SPIN Kit for Soil (MP Biomedicals) with three bead‐beating periods of 1 min 21. Bacterial DNA concentration was normalised to 1 ng/μL by dilution with DNA elution solution (MP Biomedicals, UK) to produce a final volume of 20 μL.

### 2.6 16S ribosomal RNA gene sequencing

Normalised DNA samples were sent to the Centre of Genomic Research (Liverpool, UK) for PCR amplification of the 16S ribosomal RNA (rRNA) gene, and paired‐end Illumina sequencing (2 × 250 bp) on the MiSeq platform. The V4 region of the 16S rRNA gene was amplified to generate a 254 bp insert product as described by Caporaso et al. 22. The first round of PCR was performed using the forward primer 5′‐ACACTCTTTCCCTACACGACGCTCTTCCGATCTNNNNNGTGCCAGCMGCCGCGGTAA‐3′ and the reverse primer 5′‐GTGACTGGAGTTCAGACGTGTGCTCTTCCGATCTGGACTACHVGGGTWTCTAAT‐3′, which include recognition sequences that enable a second nested PCR, using the N501f and N701r primers, to incorporate Illumina adapter sequences and barcode sequences. The use of these primers enable efficient community clustering for the length of reads obtained through Illumina sequencing, and this method also allows for high‐throughput sequencing. Sequencing data were supplied in FASTQ format with adaptors already trimmed. Sequencing data were analysed using the Quantitative Insights into Microbial Ecology (QIIME) pipeline and the Ribosomal Database Project (RDP) classifier 16S rRNA gene sequence database 23, 24. All sequences met the following criteria: read length within 200 and 1000 bp; an Illumina quality digit >0 and a minimum average quality score of 25 within a 50 bp window. ChimeraSlayer was used to filter trimmed reads for chimeric sequences, RDP classifier (version 2.10) was used for bacterial taxonomy assignment with a confidence value threshold of 50% and trimmed reads clustered into operational taxonomic units (OTUs) at 97% identity level. Weighted and unweighted UniFrac distances were used to generate beta‐diversity principal coordinates analysis plots, which were visualised using the Emperor tool.

### 2.7 Statistical analysis

Data from one of the 68 faecal samples was excluded from further analysis after interrogation of the sequencing data indicated that it had a poor sequencing depth (18 054 reads). This sample was collected at the end of an intervention phase, leaving only nine individuals with a complete set of pre–post‐intervention measurements (*n* = 9).

Associations between bacterial population proportions and the dietary intervention were investigated using sequential ‘type 1’ analysis of variance (ANOVA). The proportions were log transformed following the addition of a small offset (0.00001) to counteract the presence of zero values. The response variable was the transformed post‐intervention bacterial proportion, and the four explanatory variables were, respectively: the transformed pre‐intervention bacterial proportion (continuous); the participant code (*n*‐level categorical, where *n* is the number of participants); the study phase (two‐level categorical, first intervention and second intervention) and the dietary intervention identifier (two‐level categorical, high‐*Brassica* and low‐*Brassica* diets). Carry‐over was investigated by comparing the sum of the two post‐intervention measurements in the two arms of the study (high–low versus low–high).

#### 2.7.1 Multivariate analysis

A subset of 66 bacterial taxa were selected, from a total of 385 identified, based on their detection in a minimum of 45 of the 67 faecal samples. This subset accounted for >90% of the total bacterial population in 64 of 67 faecal samples. The data were log transformed, after adding a small offset (0.00001), and unit‐variance scaled. Fourteen principal components were retained based on the Kaiser criterion. A varimax rotation was applied to the retained components, and the new scores rescaled so the corresponding loadings were of unit length. The correlations between the first and second rotated factor scores and each of the log‐transformed variates were plotted to identify bacterial taxa associated with the first rotated factor scores. Associations with the dietary intervention and these bacteria were investigated using sequential ANOVA as described above. The abundance of bacteria with significance levels below a threshold determined by controlling the false discovery rate to *q* = 0.05 were considered to be associated with the dietary intervention. Statistical analysis was performed using Matlab (version 8.5), with the Statistics and Machine Learning Toolbox (version 10.0).

## 3 Results

### 3.1 Participant and dietary information

Recruitment took place between September 2014 and January 2015, with the trial ending in April 2015 due to the successful completion of the study by all ten participants. The age of the ten subjects participating in the dietary intervention study (four men and six women) ranged between 25 and 41 years, with an average age (±SD) of 33.5 ± 5.9 years (Table 1). The average body weight and BMI (±SD) of the participants at the eligibility screening assessment was 73.4 ± 13.4 kg and 24.5 ± 2.6 kg/m^2^, respectively. All participants were Caucasian, and nine of the ten participants were non‐smokers, with the remaining participant classified as an irregular smoker. Each participant consumed a low‐ and high‐*Brassica* diet in a randomised order, with the low‐*Brassica* diet supplementing the participants’ habitual diet with 4.51 mmol total sulphur and 0.11 mmol sulphate across the 2‐wk period, compared to 335.88 mmol total sulphur and 7.02 mmol sulphate during the high‐*Brassica* diet phase (Table 2). No adverse effects were reported during the study by any of the participants.

**Table 2:** Total sulphur and sulphur‐containing metabolites in cauliflower (84 g portion), broccoli (84 g portion) and broccoli and sweet potato soup (300 g portion) (mean ± SD), and the total amounts in the 2‐wk low‐ and high‐*Brassica* diets

	Mean mmol/portion			
Food	Total sulphur	Sulphate	Total glucosinolates	SMCSO
Cauliflower	0.95 (n/a)	0.09 (± 0)	0.05 (± 0.01)	0.22 (± 0.01)
Broccoli	3.56 (n/a)	0.02 (± 0)	0.11 (± 0.01)	0.23 (± 0.01)
Broccoli and sweet potato soup	51.47 (± 0.98)	1.06 (± 0.04)	0.29 (± 0.01)	1.51 (± 0.03)
Diet	Mean mmol/diet			
Low‐*Brassica*	4.51	0.11	0.16	0.45
High‐*Brassica*	335.88	7.02	2.70	11.76

SMCSO, *S*‐methylcysteine sulphoxide; n/a, value obtained from single sample.

### 3.2 Effect of high‐*Brassica* diet on intestinal lactobacilli

Taxonomic identification of the 16S rRNA gene sequences was performed using QIIME and the RDP classifier. The resultant data did not support the hypothesis that the increased consumption of *Brassica* vegetables would be associated with a change in the relative abundance of members of the genus *Lactobacillus* (*p* = 0.8019).

### 3.3 Association between *Brassica* diets and SRB

**Figure 1:**
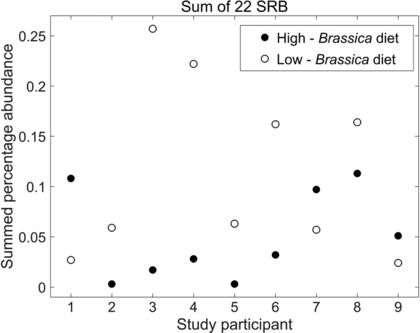
Increased consumption of *Brassica* is associated with reduced relative proportions of sulphate‐reducing bacteria in the human gut microbiota. Summed percentage abundance of total sulphate‐reducing bacteria in the human gut microbiota following a 2‐wk low‐ or high‐*Brassica* diet. Bacterial DNA was extracted from faecal samples, and the 16S rRNA genes were sequenced using paired‐end sequencing on an Illumina MiSeq platform. Bioinformatic analysis was performed using QIIME 1.8.0 and RDP classifier. Data from participant 10 are not shown due to poor sequencing depth in the sample obtained following the low‐*Brassica* diet.

Interrogation of the 16S rRNA dataset revealed the populations of SRB within the gut microbiota of participants. Twenty‐two taxa corresponding to SRB were identified, with members of the *Desulphovibrionaceae* present in all but one of the faecal samples obtained from each individual. Bacteria of the genera *Bilophila* and *Desulphovibrio* were most highly represented in the 67 faecal microbiota samples at an average of 0.049% and 0.033% of the total population, respectively, with *Bilophila* detected in 97% of the samples. The relative proportions of total SRB were observed to range between <0.01% and 0.46% of the total population of bacteria present in each of the 67 faecal samples obtained throughout the study, with a geometric mean of 0.056% of the total bacterial population across all 67 faecal samples (additional data in Supporting Information Table 1). The 16S rRNA gene sequencing data were investigated to identify whether consumption of the high‐*Brassica* diet led to a proportional increase of SRB within the gut microbiota of participants. There was moderate evidence (*p* = 0.0215) that an increase in the consumption of *Brassica* is associated with a reduction in the abundance of SRB, as was seen in six of the nine participants tested (Fig.1). Interestingly, the three participants that showed the reverse pattern had the highest BMI values.

### 3.4 Beta‐diversity analysis of faecal microbiota composition

**Figure 2:**
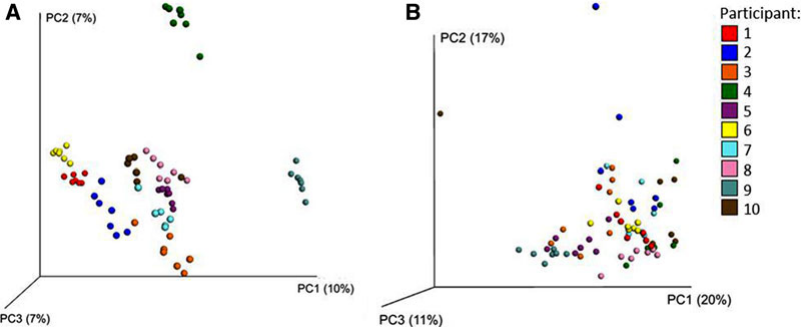
Beta‐diversity analysis shows clustering of faecal microbiota based on the individual. Beta‐diversity analysis of faecal microbiota samples from ten study participants; each participant collected four to five non‐intervention samples, a single sample following a 2‐wk low‐*Brassica* diet and a single sample following a 2‐wk high‐*Brassica* diet. (A) Unweighted beta‐diversity analysis; (B) weighted beta‐diversity analysis. Beta‐diversity analysis was performed using the UniFrac metric using QIIME 1.8.0, and visualised as a 3D principal coordinates analysis plot using Emperor. The code in the key refers to the different participants.

The bacterial DNA was extracted from the faecal samples, and the V4 region of the 16S rRNA gene were amplified using PCR, and sequenced using the paired‐end Illumina MiSeq platform, for downstream analysis using the QIIME pipeline. This produced 6 503 134 high‐quality reads, with an average of 95 634 ± 28 729 reads per sample, which clustered into 21 563 operational taxonomic units at 97% identity. Bioinformatic analysis was performed on the sequencing data obtained from the faecal samples of all ten participants, however a single sample was excluded from further analysis due to poor sequencing depth (18 054 reads). Although consumption of either of the *Brassica* diets did not lead to a noticeable common alteration of the gut microbiota composition, it was observed that samples obtained from each participant clustered together (Fig. 2). This analysis indicated that the community structure of the gut microbiota was unique to each participant, and that the relative abundance of bacteria within these communities may have fluctuated over time, which would allow for a degree of plasticity within the microbiota.

#### 3.4.1 Multivariate analysis of faecal microbiota composition

**Figure 3:**
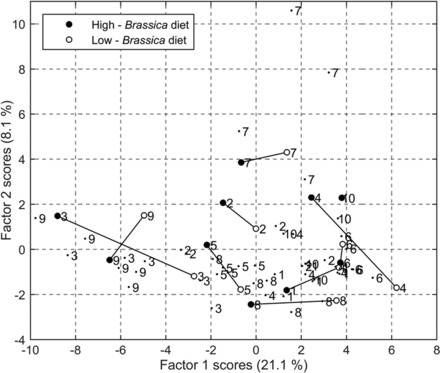
Variates corresponding to 66 bacterial taxa within faecal samples collected after the high‐*Brassica* diet are associated with a lower first factor score than those collected after the low‐*Brassica* diet. Score plot of the first two varimax‐rotated factors: each point represents the scores of the subset of 66 bacterial taxa. Black dots relate to non‐intervention faecal samples, closed circles represent faecal samples collected after the high‐*Brassica* diet and open circles denote faecal samples collected after the low‐*Brassica* diet. Scores labelled with the same number (1–10) correspond to a single individual, and scores obtained for the low‐ and high‐*Brassica* diet for each individual are connected by a black line. As the data from the sample collected after the low‐*Brassica* diet for participant 10 were excluded, no connecting line is present for the samples of this participant.

**Figure 4:**
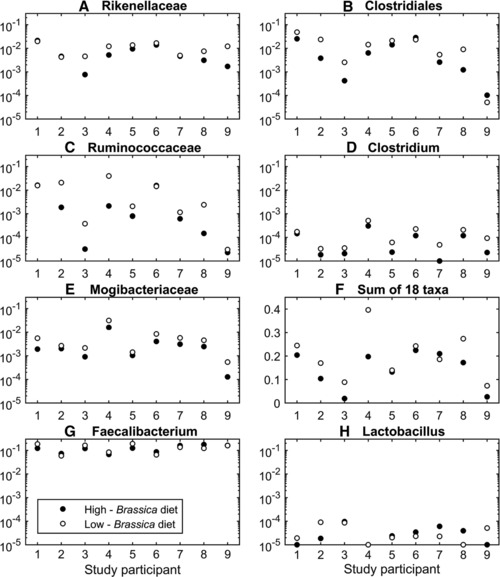
Decreased proportions of the strongly significant members of the bacterial taxa associated with the *Brassica* diets (*p* < 0.01) (A–E) are observed in most of the nine participants tested after consumption of the high‐*Brassica* diet. All subplots represent the relative population proportions of bacteria within the human faecal microbiota of samples obtained from nine participants after the high‐*Brassica* (filled circles) and low‐*Brassica* (open circles) diets. (A–E) Members of the five bacterial taxa significantly associated with consumption of the *Brassica* diets (*p* < 0.01). (F) The summed proportions of the 18 bacterial taxa positively associated with the first varimax‐rotated factor. (G, H) For comparison, relative proportions of the genera *Faecalibacterium* and *Lactobacillus*, respectively, are shown as no statistically significant dietary effects were observed. In all subplots except (F), the proportions are presented on a log scale following the addition of a small offset (0.00001). The plots were produced using Matlab.

Multivariate analysis was performed on the bacterial taxa that were detected in a minimum of 45 of the 67 faecal microbiotas. This criterion found 66 of the total 385 bacterial taxa as candidates for further analysis, with this subset accounting for >90% of the total population in 64 of 67 faecal microbiotas. The data were log transformed and variance scaled, and 14 principal components were retained based on the Kaiser criterion. A varimax rotation was performed on the 14 principal components to produce the factor analysis scores. This transformation aims to find scores that are strongly associated (correlated) with subsets of bacterial taxa. A score plot of the first and second varimax rotated factor scores highlighted a trend in which the bacterial community profile summarised by the first factor produced scores that were lower after participants consumed the high‐*Brassica* diet than after consumption of the low‐*Brassica* diet (Fig. 3). Nineteen bacterial taxa with a strong association with the first factor score were selected for subsequent ANOVA, of which 18 were negatively associated with a high‐*Brassica* diet. Comparison of the two study arms showed little evidence of carry‐over effects (maximum significance level, *p* = 0.0966). After controlling the false discovery rate to *q* = 0.05, there was evidence that the abundance of ten of the 19 selected taxa were associated (*p* ≤ 0.05) with the dietary intervention, with five of these taxa exhibiting a strong association (*p* ≤ 0.0078; emboldened in Table 3). The data displayed in Table 3 indicate that the relative proportions of all presented taxa had decreased after the high‐*Brassica* diet, whilst most (8/10) increased after the low‐*Brassica* diet. Figure 4A–E displays the log‐transformed proportions of the five bacterial taxa with *p* < 0.01 after each of the *Brassica* dietary interventions for the nine participants analysed. Of these, four taxa were identified as members of the order *Clostridiales*, with the remaining taxon belonging to the family *Rikenellaceae*. Figure 4F shows the summed proportions of the 18 bacterial taxa positively associated with the first factor score, after each of the *Brassica* dietary interventions. Figure 4G shows that there is little evidence of a difference in the proportions of the genus *Faecalibacterium*, a prominent and important member of the human gut microbiota, following the consumption of either *Brassica* diet (*p* = 0.8473).

**Table 3:** Bacterial taxa selected by the multivariate analysis with strengths of association with the dietary intervention, *p* < 0.05

Bacterial taxa	Low‐*Brassica* diet						High‐*Brassica* diet						Day 15 high to Day 15 low		
	Day 0		Day 15		Diff		Day 0		Day 15		Diff		Mean	SD	*P*
	Mean	SD	Mean	SD	Mean	SD	Mean	SD	Mean	SD	Mean	SD			
p_Firmicutes	−7.15	2.17	−6.88	1.71	0.26	0.88	−7.41	1.41	−7.80	1.74	−0.38	1.11	−0.91	1.04	0.0206
o_*Clostridiales*	−5.02	2.12	−4.93	2.05	0.09	0.68	−5.10	1.56	−5.75	1.89	−0.64	1.29	−0.82	0.93	**0.0037**
f_*Ruminococcaceae*	−6.35	2.34	−5.86	2.29	0.49	0.80	−6.95	2.34	−7.23	2.38	−0.28	0.79	−1.37	1.26	**0.0001**
f_*Mogibacteriaceae*;other	−7.83	1.65	−7.90	1.81	−0.07	0.84	−8.31	1.61	−8.60	1.76	−0.29	0.82	−0.70	0.77	0.0360
f_*Mogibacteriaceae*;g	−5.86	1.31	−5.57	1.15	0.29	0.49	−5.99	1.15	−6.31	1.30	−0.31	0.31	−0.74	0.36	**0.0024**
f_*Rikenellaceae*;other	−6.17	1.00	−5.76	0.72	0.42	0.79	−6.24	1.18	−6.58	1.39	−0.33	1.40	−0.82	1.03	0.0295
f_*Rikenellaceae*;g	−5.06	0.87	−4.69	0.59	0.38	0.83	−5.09	1.14	−5.35	1.03	−0.26	1.02	−0.66	0.77	**0.0078**
g_*Alistipes*	−7.85	1.45	−7.28	1.31	0.57	0.76	−8.29	1.44	−8.38	1.60	−0.09	1.51	−1.10	0.88	0.0254
g_*Dehalobacterium*	−8.84	1.76	−8.72	1.90	0.12	0.52	−9.46	1.79	−9.42	1.79	0.04	1.18	−0.69	0.86	0.0206
g_*Clostridium*	−8.98	0.83	−9.17	0.96	−0.20	1.07	−9.42	0.87	−9.94	1.19	−0.53	0.70	−0.77	0.45	**0.0059**

Day 0 and Day 15, values obtained before and after each dietary intervention phase; Diff, paired differences between values obtained before and after each dietary intervention phase; Day 15 high to Day 15 low, paired differences between values obtained after the high‐ and low‐*Brassica* diet intervention phases; *p*, significance of *Brassica* diet in analysis of variance model, with bold values indicating *p* ≤ 0.0078. Bacterial proportions expressed as the natural logarithm of the fractional proportion of population after the addition of a small offset (0.00001). p, phylum; o, order; f, family; g, genus. Comparisons based on data from nine individuals (*n* = 9).

## 4 Discussion

We report the results of a randomised crossover study to investigate whether a 2‐wk diet rich in *Brassica* vegetables would be associated with an increase in members of the intestinal lactobacilli or SRB, or cause other perturbations to the colonic microbiota. Participants were Caucasians, and included both sexes and a range of ages and BMI values. *Brassica* vegetables are a rich source of fibres and vitamins, and contain relatively high amounts of sulphur‐containing compounds and inorganic sulphate. Florin et al. reported that ∼0.22 g of dietary sulphate per day can be absorbed in the small intestine, with any additional dietary sulphate reaching the colon where it would be available for utilisation by SRB 25. Therefore, an increase in the consumption of *Brassica* vegetables may adversely affect the levels of SRB in the GI tract.

This study found that the *Brassica*‐rich diet was not associated with an increase in lactobacilli, but was associated with a decrease in the relative proportions of SRB, compared to the low‐*Brassica* diet (Fig. 1). Twenty‐two bacterial taxa with the ability to utilise sulphate, thiosulphate or sulphite were detected in the faecal samples, ranging from <0.01 to 0.46%, and with a geometric mean of 0.056% of the total population over the 67 faecal samples in which SRB were detected. Although the observed taxa varied between individuals, members of *Desulphovibrio* and *Bilophila* (a close relative of *Desulphovibrio* species 26) were present in 97% of the faecal samples tested. This agrees with previous studies that reported *Desulphovibrio* species as the principal SRB found in the human GI tract 27, 28, 29.

A high‐protein diet has been reported to lead to proportional increases in SRB and faecal sulphide 30, which have been linked with an increased likelihood of relapse in ulcerative colitis patients 31, 32. Conversely, a study investigating the effects of inorganic sulphate‐supplemented drinking water in mice did not find significant increases in intestinal sulphate or H_2_S concentration, suggesting no significant increase in SRB activity 33. The authors postulated that inorganic sulphate (as is found in *Brassica* vegetables) was not a determining factor in H_2_S production associated with SRB, and that H_2_S generated from sulphur‐containing amino acid dissimilation may be primarily responsible for the majority of intestinal H_2_S 33. It was also reported that increased supplementation of drinking water with inorganic sulphate correlated with an increased density of sulphomucin‐containing goblet cells in mice, linking dietary inorganic sulphate with an increase in the sulphation of mucins within the large intestine. The results of this study suggest that the high consumption of *Brassica* vegetables reduced the abundance of SRB. Further studies could examine whether a high‐*Brassica* diet may also be associated with a regeneration of the human intestinal mucosa.

In addition to quantifying changes in the relative proportions of intestinal lactobacilli and SRB, the effect of a high‐*Brassica* diet on gut microbiota composition was investigated. Phylogenetic analysis of the faecal microbiota suggested the bacterial communities clustered by participant, indicating the unique nature and inherent stability of an individual's gut microbiota (Fig. 2A). Inter‐individual differences in the composition of gut bacterial communities have also been observed in other studies 4, 34, 35. Weighted beta‐diversity analysis suggested the microbiota of each individual clustered, but that there was variation in the abundances of bacteria within the communities during the study period (Fig. 2B). The variation of bacterial abundances within individuals may reflect the plasticity of the communities. However, the use of faecal samples to measure gut bacterial communities can introduce bias due to differences between aliquots of a single sample, which we attempted to avoid by homogenising the sample first. Li et al. reported changes to the composition of human gut bacterial following a ‘double‐cruciferous diet’ (basal diet + 14 g cruciferous vegetables/kg body weight per day) versus the basal diet 4. As the basal diet was devoid of vegetables, fruits, whole grains and high‐fibre foods, the addition of a large portion of cruciferous vegetables would provide a range of nutrients, which the bacterial community may not have been able to obtain from other dietary sources. In contrast, in the current study participants were only required to restrict their diet of *Brassica* vegetables and other glucosinolate‐containing foods.

While analysis of the bacterial communities with the use of the Unifrac metric indicated the *Brassica* diets were not associated with a change at the community level (Fig. 2), an exploratory multivariate approach suggested the relative abundances of certain bacterial taxa were perturbed by the dietary intervention. Relative proportions of five bacterial taxa—four members of the *Clostridiales* (*Ruminococcaceae*, *Mogibacteriaceae*, *Clostridium*, an unclassified *Clostridiales*) and one member of the *Bacteroidales* (*Rikenellaceae*)—were lower following the consumption of the high‐*Brassica* diet, compared to the low‐*Brassica* diet (*p* ≤ 0.0078) (Table 3). Li et al. also reported changes in the frequency of members of the *Clostridiales* and *Rikenellaceae*, but did not report the direction of change with a high cruciferous diet 4.

It is unclear why the high‐*Brassica* diet was associated with a decrease in the relative proportions of the members of the bacterial taxa observed, but one explanation may be an inhibitory effect caused by relatively high concentrations of sulphur‐containing compounds. Filocamo et al. reported an inhibitory effect of garlic powder on pure cultures of *Clostridium nexile* 36, and it is possible that other members of the *Clostridiales* may be sensitive to dietary sulphur‐containing compounds.

A potential limitation of this study was the use of 16S rRNA gene sequencing to characterise the faecal microbiota of the participants. Whole genome shotgun sequencing would have enabled a more robust classification at the species level and enhanced detection of bacterial diversity 37.

In summary, it was shown that a *Brassica*‐rich diet did not significantly alter the relative proportions of intestinal lactobacilli, but was associated with a reduction in the relative abundances of SRB, as well as systematic changes in the relative abundances of certain bacterial taxa. Further studies are needed to gain more information on the metabolic fate of sulphate from *Brassica* vegetables, and to clarify the effects of *Brassica* consumption on members of the gut microbiota. However, the results from this study indicate that a diet rich in *Brassica* vegetables would not be expected to be associated with GI complaints linked to increased levels of SRB.

*The authors had no conflicts of interest to declare*.

## Supporting information


**Supplementary Table 1**. Summed percentage relative abundance of total sulphate‐reducing bacteria in all 67 of the human faecal samples collected from the 10 participants during the study period. With the exception of the ‘pre study’ sample that was collected within 24hrs before the study started, all other samples were collected after a minimum of 2wks. Of the potential 70 samples, one sample from participant 10 did not meet the required sequencing depth (as described in main manuscript, section 3.4), and two of the study periods are identical for two participants (participant 7 = ‘post low diet’ period and the ‘pre high diet’ period, participant 10 = ‘pre low diet’ period and the ‘post high diet’ period) resulting in a total of 67 samples.
