# Supplementary material for: Consumption of a diet rich in Brassica vegetables is associated with a reduced abundance of sulphate‐reducing bacteria: A randomised crossover study
Source: Mol Nutr Food Res. 2017 Apr 12;61(9):1600992. doi: 10.1002/mnfr.201600992 (PMC5600105; doi:10.1002/mnfr.201600992)
Supplement: Supplementary file 1 — Supplementary Table 1. Summed percentage relative abundance of total sulphate‐reducing bacteria in all 67 of the human faecal samples collected from the 10 participants during the study period. With the exception of the ‘pre study’ sample that was collected within 24hrs before the study started, all other samples were collected after a minimum of 2wks. Of the potential 70 samples, one sample from participant 10 did not meet the required sequencing depth (as described in main manuscript, section 3.4), and two of the study periods are identical for two participants (participant 7 = ‘post low diet’ period and the ‘pre high diet’ period, participant 10 = ‘pre low diet’ period and the ‘post high diet’ period) resulting in a total of 67 samples. [file MNFR-61-na-s001.docx]

Supplementary material

The list of foods that participants were asked to avoid during the study period:

- **Mustard** : all types including ethiopian mustard, indian mustard, chinese mustard, red giant mustard, wrapped heart mustard cabbage, yellow mustard, black mustard, broad beak mustard, purple stem mustard, mustard spinach.
- **Broccoli:** all types including rapini or broccoli raab, alboglabra kai-ian (chinese broccoli), romanesco broccoli, broccoli, broccoflower.
- **Sprouting broccoli**
- **Brussels sprouts**
- **Cabbage:** all types including white cabbage (drum), head cabbage, savoy cabbage, red cabbage, green cabbage.
- **Kale:** all types including curly kale, chinese kale, sea kale, pabularia siberian kale, acephala Kale.
- **Kohl rabi**
- **Rutabaga:** Swede, neep, turnip and turnip tops.
- **Collard greens and spring greens**
- **Chinese cabbage; Pak Choi; bok choi** and other Chinese brassica vegetables.
- **Radish**
- **Salad rocket**
- **Horseradish**: sauces and vegetables.
- **Cress:** all types, including watercress and garden cress.
- **Papaya seeds**
- **Cauliflower**
- **Wasabi**

**Supplementary** **Table 1.** Summed percentage relative abundance of total sulphate-reducing bacteria in all 67 of the human faecal samples collected from the 10 participants during the study period**.** With the exception of the ‘pre study’ sample that was collected within 24hrs before the study started, all other samples were collected after a minimum of 2wks. Of the potential 70 samples, one sample from participant 10 did not meet the required sequencing depth (as described in main manuscript, section 3.4), and two of the study periods are identical for two participants (participant 7 = ‘post low diet’ period and the ‘pre high diet’ period, participant 10 = ‘pre low diet’ period and the ‘post high diet’ period) resulting in a total of 67 samples.

| **Participant code** | **Pre study** | **Pre low diet** | **Low diet** | **Post low diet** | **Pre high diet** | **High diet** | **Post high diet** |
| --- | --- | --- | --- | --- | --- | --- | --- |
| **1** | 0.022 | 0.009 | 0.027 | 0.027 | 0.103 | 0.108 | 0.067 |
| **2** | 0.045 | 0.115 | 0.059 | 0.182 | 0.028 | 0.003 | 0.046 |
| **3** | 0.028 | 0.076 | 0.257 | 0.041 | 0.041 | 0.017 | 0.234 |
| **4** | 0.043 | 0.195 | 0.222 | 0.074 | 0.046 | 0.028 | 0.018 |
| **5** | 0.010 | 0.023 | 0.063 | 0.086 | 0.027 | 0.003 | 0.085 |
| **6** | 0.202 | 0.106 | 0.162 | 0.136 | 0.119 | 0.032 | 0.086 |
| **7** | 0.099 | 0.051 | 0.057 | 0.087 | 0.087 | 0.097 | 0.059 |
| **8** | 0.031 | 0.190 | 0.164 | 0.076 | 0.092 | 0.113 | 0.050 |
| **9** | 0.008 | 0.006 | 0.024 | 0.006 | 0.048 | 0.051 | 0.057 |
| **10** | 0.342 | 0.180 | No data | 0.223 | 0.460 | 0.135 | 0.180 |
